# Personalized blood glucose prediction: A hybrid approach using grammatical evolution and physiological models

**DOI:** 10.1371/journal.pone.0187754

**Published:** 2017-11-07

**Authors:** Iván Contreras, Silvia Oviedo, Martina Vettoretti, Roberto Visentin, Josep Vehí

**Affiliations:** 1 Institut d’Informàtica i Aplicacions, Parc Científic i Tecnològic de la Universitat de Girona, Girona, Spain; 2 Department of Information Engineering, University of Padova, Padova, Italy; University of Colorado Denver School of Medicine, UNITED STATES

## Abstract

The large patient variability in human physiology and the effects of variables such as exercise or meals challenge current prediction modeling techniques. Physiological models are very precise but they are typically complex and specific physiological knowledge is required. In contrast, data-based models allow the incorporation of additional inputs and accurately capture the relationship between these inputs and the outcome, but at the cost of losing the physiological meaning of the model. In this work, we designed a hybrid approach comprising physiological models for insulin and grammatical evolution, taking into account the clinical harm caused by deviations from the target blood glucose by using a penalizing fitness function based on the Clarke error grid. The prediction models were built using data obtained over 14 days for 100 virtual patients generated by the UVA/Padova T1D simulator. Midterm blood glucose was predicted for the 100 virtual patients using personalized models and different scenarios. The results obtained were promising; an average of 98.31% of the predictions fell in zones A and B of the Clarke error grid. Midterm predictions using personalized models are feasible when the configuration of grammatical evolution explored in this study is used. The study of new alternative models is important to move forward in the development of alarm-and-control applications for the management of type 1 diabetes and the customization of the patient’s treatments. The hybrid approach can be adapted to predict short-term blood glucose values to detect continuous glucose-monitoring sensor errors and to estimate blood glucose values when the continuous glucose-monitoring system fails to provide them.

## Introduction

The human body requires the maintenance of blood glucose (BG) levels in a very narrow range (70–110 mg/dl). Many exogenous factors affect these levels. The pancreas releases insulin and glucagon hormones secreted by β-cells and α-cells, respectively, to regulate the BG levels. Type 1 diabetes mellitus (T1D) is the consequence of an autoimmune attack on β-cells that significantly impairs insulin production. Thus, individuals with T1D fully rely on external insulin to manage their BG levels.

Therapies based on continuous glucose monitoring (CGM) devices associated to insulin pump technology (combined systems CGM-CSII) are rapidly becoming more common. A key part of these therapies in order to be truly effective is a decision support system, or an artificial pancreas that is able to predict what is going to happen in a relatively long period of time.

As widely known in clinical practice, achieving tight glycemic control is a complex process for certain patients who exhibit large variations in their BG signals due to several factors that influence the glycemic response and thereby influence glycemic control. Factors such as physical activity, weather conditions, dietary disturbances, age, and the psychological state of the patient [1][2][3] in conjunction with endogenous processes such as circadian rhythms [2], other diseases, and the menstrual period and pregnancy in women [4][5] strongly affect glucose metabolism. Because these factors are varied and often not easily identifiable, the prediction of BG values using personalized models is particularly important. Personalized models can capture specific lifestyle factors that influence the physiological response of each patient to carbohydrate intake and insulin dosage. The great variation in the glycemic response of T1D patients makes predictive modeling a challenging and crucial task.

The treatment of diabetes is conditioned by high intra- and inter-patient variability. Inter-patient variability greatly limits the use of general models because they cannot capture the specific physiological behavior of an individual. Intra-patient variability makes it difficult to apply one model for the glucose dynamics of an individual. Inter- and intra-patient variability is tackled by personalizing and customizing prediction models. This study avoids the limitations of classical modeling by implementing a set of customized models for each patient using an evolutionary approach. This paper targets the midterm (120 min) anticipated BG level while considering the clinical safety of the predictions. The models are based on a machine-learning algorithm that is flexible enough to include innovative features.

Most recent studies on BG prediction used only data-driven models [4][5] or a complementary approach that combined data-driven models and compartmental models [6][7]. Other works focused on control applications for predictions, such as the prevention of nighttime hypoglycemia [8].

BG prediction models are classified into three types: physiological, data-driven, and hybrid. Physiological models require a good understanding of insulin and glucose metabolism and contain parameters that should be set only by those with expert knowledge. These models are commonly used in simulators via compartmental models, as discussed in [9]. Minimal versions of some physiological models exist [10][11]; however, the main challenge of this type of approach is achieving a good model with high generalization capability. Data-driven models completely rely on BG data and possibly other inputs. Data-driven models are typically based on machine learning techniques and use techniques such as genetic algorithms, robust filters, fuzzy logic, rule-based models [12], multi-model approaches [13][14], autoregressive models [15][16], regularized learning, reinforcement learning, random forests, support vector regression, and artificial neural networks models [17]. Finally, an alternative architecture for BG prediction models involves properly setting a physiological model to describe glucose digestion and absorption, a second model for insulin absorption, and possibly other models to account for exercise or other events. These models constitute a preprocessing stage, the output of which enters a data-driven model. This type of model is commonly known as a hybrid model and some recent approaches to them were examined in previous studies [18][19][20]. Oviedo et al. [21] provided a comprehensive review of models for predicting BG.

Recently, BG estimation using grammatical evolution (GE) was included in a study [22] in which a novel customization of BG models for five virtual patients using GE was proposed. GE is a search algorithm with a modular design that can be used to generate predictive time series models. It uses an evolutionary-like process to achieve expressions or computer programs optimized according to a predefined objective function. GE uses a grammar to implement a linking process between the search algorithm and the actual solution. This is a key component of GE and one of the reasons why GE is attractive. The grammar consists of a set of rules that defines the structure of the expressions generated and thus the final solution by the algorithm. This structure can be modified fairly easily according to the applications needs, meaning that it can be as simple or as complex as the user determines, without altering the search algorithm performance. Authors in [22] incorporated medical knowledge into a grammar aimed to build expression for glucose that considered previous BG values, carbohydrate intake, and insulin administration. This incorporation involved exploring four different grammars and five fitness functions, all of which were evaluated with respect to average error as a performance metric for all patients. The results indicated that it is feasible to evolve useful models for modeling BG values that consider BG readings, meals, and insulin dose information. Another study [23] extended the findings of [22] by including three additional virtual patients and using the root-mean-square error (RMSE) as the fitness function. The authors tested the clinical significance of the results using error grid analysis (EGA) via the Clarke error grid (CEG) and the Parkes error grid.

The present study extends the aforementioned research to investigate a novel and complementary approach that uses symbolic regression through GE to determine an approximation of the underlying glucose dynamics evolving personalized BG predictive models that incorporate physiological models as part of the input. The aim of this approach is to capture the particular lifestyle factors that influence the physiologic response of T1D patients to their insulin doses and carbohydrate intakes. Thus, the study presents a tool meant to assist in T1D management issuing early warnings related to ineffective or poor treatments that could improve overall health, safety, and the quality of life of T1D patients.

The paper is organized as follows: Section 2 describes the type of data sets that are collected and the specific algorithms used to achieve the proposed tasks. Section 3 presents a summary of the experiments and results obtained. Section 4 discusses the results and compares and contrasts this approach with that of other works. Section 5 concludes the paper with a brief summary of the study and a discussion of future challenges.

## Materials and methods

**Fig 1:**
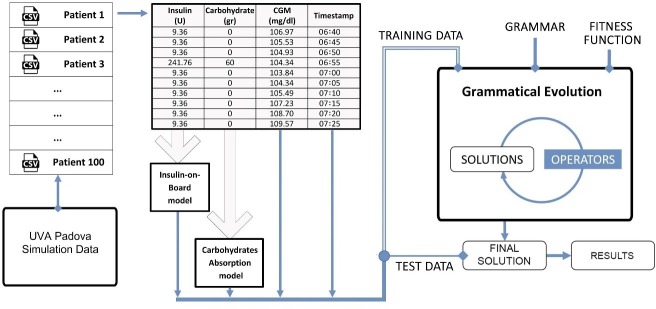
Schematic representation of the method used to generate prediction models for BG values.

Fig 1 shows a schematic representation of the methodology proposed in this study. Initially, insulin, carbohydrate, and CGM data for 100 virtual subjects are generated by simulation over 14 d using the T1D patient decision-making model [24]. The information is preprocessed and divided into files, one file per patient. The information on this files regarding the carbohydrates consumption in each meal is transformed into a continuous signal according to a physiological model that describes the absorption of the carbohydrates. Additionally, the bolus and basal insulin are added and a single continuous insulin signal is generated using an insulin-on-board model. The use of these two physiological models in this stage is beneficial not only because it smooths the original data but also because it provides values proportional to the actual behavior of the diabetic patient. Therefore, the input of the proposed GE-based tool is the output of the aforementioned physiological models in conjunction with a glucose specific fitness function and a customized grammar that represents a flexible structure, so the final solution is able to capture the patient’s dynamics. The evolutionary process commences using 10 d of historical time series data for training. The algorithm builds and adjusts prediction models until it reaches a predefined number of generations. Once the final prediction models are generated, they are evaluated using data from the remaining historical time series data. The following subsections describe the data, the physiological models and the complete GE setup in detail.

### Experimental data set

The personalized BG prediction models were built for a cohort of 100 virtual patients, the data for which were generated using the UVA/Padova T1D simulator [9] that implemented the T1D patient decision-making model [24]. That model consists of four submodels that describe the physiology of the T1D patient, the device used for glucose monitoring, the therapeutic decisions of the patient, and the insulin pump. Specifically, T1D patient physiology is defined by the UVA/Padova T1D simulator, which is a software program approved by U.S. Food and Drug Administration (FDA) as a substitute for preclinical trials for certain insulin treatments. The simulator program is based on a mathematical model of glucose, insulin, and glucagon dynamics in T1D and is equipped with a virtual population that was proven to represent intersubject glucose variability observed in a clinical trial [25]. Recently, the simulator was updated by incorporating a model of circadian insulin sensitivity variability [26] to extend its domain of validity from single-meal to single-day multiple-meal scenarios, thus enabling more realistic in silico trials [27]. For this study, the T1D patient decision-making model was used to simulate the data sets of 100 subjects. Treatment decisions were based on the self-monitoring of blood glucose (SMBG) measurements, simulated by a model of the One Touch Ultra 2 measurement error [28], and a blinded CGM sensor, the readings of which were simulated by a model of the Dexcom G4 Platinum sensor [29]. Each virtual patient data set comprised the data of a 14-d time series of BG readings collected by the CGM sensor using a 5-min sampling period, and carbohydrate (CHO) intake and insulin delivery via an insulin pump with a 1-min sampling period.

The CHO intake (g) included the carbohydrates from three meals per day with average intakes of 50, 60, and 63.5 g for breakfast, lunch and dinner, respectively, and a coefficient of variation (CV) of 20%, sampled using a Gaussian distribution. The CHO intake time series also included 20-g hypotreatments that were generated every 20 min when the glucose concentration fell below 60 mg/dl, as indicated by the SMBG measurements.

The insulin time series was the sum of the administered basal insulin I_b_ dose and the bolus insulin I_bolus_ dose (U), expressed at each time step as I(k) = I_b_(k) + I_bolus_(k), where k indexes the current sample. This step differed from the simulations performed in a previous study [24] because time-varying basal insulin was used to follow the variability pattern of insulin sensitivity. Bolus doses administered at mealtime are calculated as follows:

$$I_{bolus}=\frac{{CHO}_{IN}}{CR}+\frac{\left(G_{T}-G_{B}\right)}{CF}$$ (1)

where *CHO*_*IN*_ is the estimated amount of CHO in the ingested meal, *CR* is the CHO-to-insulin ratio, *CF* is a correction factor, *G*_*T*_ is the glucose target, and *G*_*B*_ is the current preprandial SMBG measurement. *CHO*_*IN*_ is calculated by adding a percentage error in the count of CHO (sampled from a Gaussian distribution with zero mean and 20% CV) to the actual CHO content of the meal.

**Fig 2:**
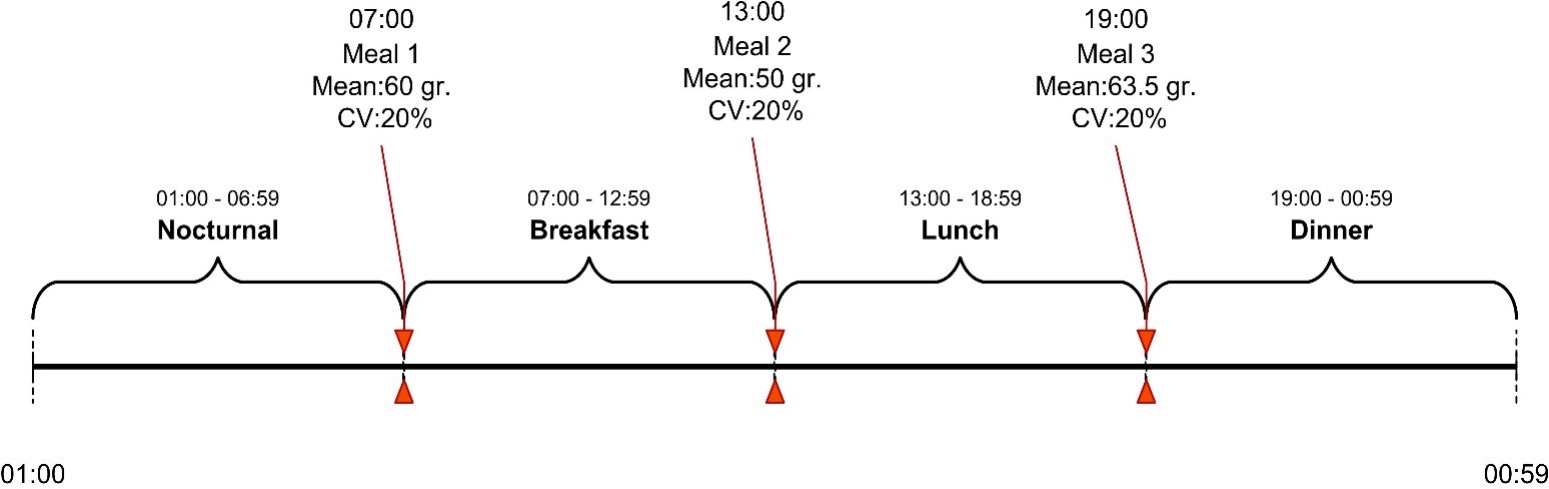
Division of the daily data for piecewise modeling.

The virtual data used in this study were subject to great variability with respect to the T1D patients. First, the time-varying factors and perturbations implemented in the simulator allowed the use of a set of virtual patients with significant intrapatient variability. Second, the 100 virtual patients used in the simulation had distinct physical characteristics, allowing for the inclusion of interpatient variability in the data set. The modeling algorithm read the data for each patient, taken over 14 consecutive days. The CGM readings (mg/dl) were used in two ways: first, as a source of information to predict future glucose values (i.e., historical data), and second, as a reference value to train or validate the models. We used a piecewise approach in which three models predicted the postprandial BG values and one model predicted the BG value for the overnight period for each patient. As shown in the timeline in Fig 2, each day was divided into four periods of 6 h. Each period contained the same amount of data and were labeled *Nocturnal* from 01:00 to 06:59 h, *Breakfast* from 07:00 to 12:59 h, *Lunch* from 13:00 to 18:59 h, and *Dinner* from 19:00 h to 00:59 h.

The midterm prediction model was developed using simple physiological models to exploit the information contained in the insulin and CHO data series to transform the inputs into continuous signals, and a data-driven model to map past values of the model inputs to future BG values. Each part is discussed in the following two subsections.

### Physiological modeling

According to a study by Hovorka et al. [30], glucose excursions are influenced by the glucose absorption process and can be represented by a two-compartment model that delivers the glucose at an absorption rate (mg/min)

Ra(t)=CHOIN*CHOBIO*t*e(−ttmax,G)tmax,G2 (2)

where *t*_max,G_ (min) is the time of the maximum appearance rate of glucose in the accessible glucose compartment, *CHO*_*IN*_ is the amount of carbohydrates ingested, and *CHO*_*BIO*_ (dimensionless) is carbohydrate bioavailability. The glucose absorption rate greatly affects the levels of BG, so the *R*_a_ signal is generated using the carbohydrate intake and population values at *t*_max,G_ = 50 min and *CHO*_*BIO*_ = 0.8.

For the insulin dose, we used the estimation of insulin on board (IOB) obtained with a two-compartment model based on [31]. Smart insulin pumps can calculate the insulin that remains active within the body by using an estimation of IOB defined as follows:

$$\begin{array}{c} \frac{dC_{1}(t)}{dt}={u(t)-K}_{DIA}C_{1}(t)\\ \frac{dC_{2}(t)}{dt}=K_{DIA}(C_{1}(t)-C_{2}(t))\\ I_{OB}(t)=C_{1}(t)+C_{2}(t) \end{array}$$ (3)

where DIA is the duration of insulin action (h), which parameterizes the model of *I*_OB_(*t*) and characterizes the dynamics of insulin activity; *C*_1_(*t*) and *C*_2_(*t*) denote the compartments, *u*(*t*) is the insulin dose, and the constant *K* replicates its corresponding DIA. For our study, we used a discrete time approximation of the model in Eq (3) with *K*_*DIA*_ = 0.039, which corresponds to a DIA of 2 h.

### Predictive modeling by GE

Grammatical evolution (GE) [32] is a population-based heuristic search algorithm that performs an evolutionary process through selection, recombination, and mutation of a rule-based rewriting sequence on variable-length binary strings. The goal of this evolutionary computational technique is to construct syntactically correct programs that can be assessed in terms of a fitness function. The key to this construction is the grammar that allows the GE to perform a genotype-phenotype mapping process, which decodes bit strings to generate programs in an arbitrary language.

At the genotype level, the underlying genetic algorithm generates and operates the population as binary strings. The genotypes are divided into a variable number of codons, with eight binary alleles representing each codon. The mapping process involves the decoding of the genotype to its phenotype, i.e., the translation of the individual codified information into a problem-specific domain. The GE approach is an attractive method because of its flexibility and because the knowledge related to the problem can be incorporated into the algorithm using a well-structured grammar. In addition, because separate approaches are used for the search and solution spaces, the phenotype can be as complex as necessary, as all the genetic operators are applied to the genotype.

The core of context-free evolutionary grammar, usually defined in Backus normal form (BNF), is a set of derivation rules expressed in the following form:

$$\left[symbol]\to \{{production}_{1}|\ldots |{production}_{N}\right\}$$ (4)

Each rule has two parts, namely, a non-terminal {symbol} on the left-hand side and a definition of the non-terminal {productions} on the right-hand side. Each definition comprises one or more alternatives separated by the symbol “|”. Each alternative is commonly called a production and is composed of a sequence of terminals (bracketless) and non-terminals. Thus, the grammar indicates that a non-terminal can be substituted for any of the defined alternatives. The grammar defines the search space of solutions; thus, the quality of the obtained solutions directly depends on this structure. The framework proposed here combines insulin, carbohydrates, and BG levels. In addition, other complex and decisive factors such as intradaily insulin sensitivity of T1D patients and the reliance of the generated models on time are considered. An excerpt summarizing the main characteristics of the defined grammar is given in Eq 5:

$$\begin{array}{c} {}[Body]\to Expr\hat{G}=(\left[G][op][R_{a}][op][I_{OB}\right])[op][Circadian];\\ {}[G]\to GetG(\left[PrevIni],[PrevFin],[op],[preop\right])[G]|\lambda \\ \left[Ra]\to GetRa(\left[PrevIni],[PrevFin],[op],[preop\right])[Ra]|\lambda \right)\\ \left[I_{OB}]\to GetIOB(\left[PrevIni],[PrevFin],[op],[preop\right])[I_{OB}]|\lambda \right)\\ {}[preop]\to sqrt|sin|log|pow|exp|cos|[preop][preop]|\lambda \\ {}[Circadian]\to GetCircadian([OpB],[Cte],[Cte],[Cte])|\lambda \\ \left[Cte]\to (\left[Dgt][Dgt].[Dgt\right]\right)\\ \left[op]\to [OpA]|[OpB\right]\\ {}[PrevIni]\to 0|1|2|4|6|8|10|12|14|16|18|20|22\\ {}[PrevFin]\to 1|2|4|6|8|10|12|14|16|18|20|22|24\\ {}[Dgt]\to 0|1|2|3|4|5|6|7|8|9\\ {}[OpA]\to +|-\\ {}[OpB]\to /|* \end{array}$$ (5)

where λ denotes the empty set that does not contain any terminals. The solutions combine four expressions, namely, (**[G]**, **[Ra]**, **[I**_**OB**_**]**, and **[Circadian]**), with four operators selected from **[Op]**. A more formal definition of these four rules in the continuous time domain can be expressed as follows:

$$\begin{array}{c} G(t)=\sum_{i=0}^{i=n}\left[preop(G_{t-\beta }^{\alpha })op\delta \right]\\ Ra(t)=\sum_{i=0}^{i=n}\left[preop({Ra}_{t-\beta }^{\alpha })op\delta \right]\\ I_{OB}(t)=\sum_{i=0}^{i=n}\left[preop({IOB}_{t-\beta }^{\alpha })op\delta \right]\\ Circadian(t)=A\;cos(\omega t+\varphi ) \end{array}$$ (6)

where **op**{+, -, *, /} and **preop**{$\sqrt{x}$, *x*^-1^, log(*x*), *x*^*y*^, and sin(*x*) cos(*x*)} denote the operators selected by the GE methodology, and ***α***, ***β***, ***δ*, *A*** (maximum elongation), ***ω*** (angular frequency), ***φ*** (initial phase), and *n* are constants that are adjusted by the GE methodology in each mathematical expression.

Despite knowing the impact of the inputs on the BG levels (i.e., insulin and carbohydrates have a negative effect on them), this knowledge is not directly incorporated into the initial rule. Instead, four basic operations are allowed to relate the expressions. The grammar is designed to constrain the search space by using functions that operate the previous values of the input signals. In addition to the operation of the three input variables (insulin, CHO, and BG), the sinusoidal function is added to account for the circadian variations in the physiology of patients in the final model (with maximum day-to-day variations having a 20% amplitude).

### Fitness function and GE setup

In the identification and predictive approaches of the BG model, the mean squared error (MSE) is the most popular loss function and metric for assessing the performance of the model:

$$MSE(g(t),\hat{g}(t,\theta ))=\frac{1}{N}\sum_{t=1}^{N}{\left(g(t)-\hat{g}(t,\theta )\right)}^{2}$$ (7)

where the parameter *θ* is selected to minimize the MSE value. MSE weights all the errors the same, even if they have different impacts in diabetes therapy. In the present study, to obtain the final model, we incorporated a fitness function based on glucose-specific MSE (gMSE), as proposed in [33]. The fitness function weights the clinical impact of errors for hypoglycemia, normoglycemia, and hyperglycemia differently. This is very beneficial to the final model since the fitness function is more sensitive to extremely harmful situations like hypoglycemia, which thereby leads to a safer model.

The fitness function used in the present study is similar to the usual quadratic MSE function. However, it includes a few additional penalties in the zones in which the error represents additional danger from a clinical perspective. For example, it is more dangerous to predict a BG concentration of 75 mg/dl when the target BG concentration actually is 50 mg/dl than it is to predict a BG concentration of 150 mg/dl when the target BG concentration actually is 175 mg/dl because missing hypoglycemic events is considerably more dangerous for the patient. The model of a glucose-specific function is

$$gMSE(g,\hat{g})=MSE(g,\hat{g})*Pen(g,\hat{g})$$ (8)

where $Pen(g,\hat{g})$ is a function that penalizes deviation based on its clinical harmfulness and is expressed as

$$Pen(g,\hat{g})=1+\alpha_{L}{\bar{\alpha }}_{g\leq T_{L},\beta_{L}}(g)\alpha_{\hat{g}\geq g,\gamma_{L}}(\hat{g},g)+\alpha_{H}\alpha_{g\geq T_{H},\beta_{H}}(g){\bar{\alpha }}_{\hat{g}\leq g,\;\gamma_{H}}(\hat{g},g)$$ (9)

where

$$\alpha_{L}=1.5,\;\alpha_{H}=1,\;\beta_{L}=30,\beta_{H}=100,\gamma_{L}=10,\gamma_{H}=20,\;T_{L}=85,T_{H}=155$$

As demonstrated in a previous study [33], the standard performance metrics are adapted using the Pen function. Therefore, we modified the fitness function using the penalization factor. This metric was also included in the report on the fitting and test deviation from the target values, as explained in the Results section.

Next, we examined the implementation of the evolutionary algorithm based on a open GE Java implementation [34]. In our approach, we used the following classic operators: elitism, variable crossover by a single point, integer flip mutation, and selection by tournament. The operator parameters are listed in Table 1. Customized genetic operators are not required in GE because it uses the standard operators of genetic algorithms [35]. The individuals are initialized randomly to generate variable-length binary strings.

**Table 1:** General parameters of the implementation of GE and its operators.

Parameters	Value	Parameters	Value
Population	50	Tournament Size	2
Generations	2000	Max. Wraps	2
Crossover prob.	0.90	Mutation prob.	0.005
Elitism	2		

## Results

### Performance metrics

The aim of this study was the production and assessment of personalized models for 100 virtual patients using the information contained in CGM readings, insulin dosage, and carbohydrate intake. This section presents the prediction results in terms of the usual performance metrics used to evaluate predictive accuracy and glucose-specific metrics based on the RMSE, the mean absolute deviation (MAD), and the mean absolute relative difference (MARD), as proposed in [33]. We present below the equations used to calculate the performance metrics.

$$\begin{array}{c} RMSE=\sqrt{\frac{1}{N}\sum_{t=1}^{N}{\left|g(t)-\hat{g}(t)\right|}^{2}}\\ gRMSE\left(\frac{mg}{dl}\right)=\sqrt{\frac{1}{N}\sum_{t=1}^{N}{Pen(g(t),\hat{g}(t))|g(t)-\hat{g}(t)|}^{2}}\\ MAD(\%)=\frac{1}{N}\sum_{t=1}^{N}\left|g(t)-\hat{g}(t)\right|\\ gMAD(\%)=\frac{1}{N}\sum_{t=1}^{N}Pen(g(t),\hat{g}(t))|g(t)-\hat{g}(t)|\\ MARD(\%)=\frac{1}{N}\sum_{t=1}^{N}\frac{\left|g(t)-\hat{g}(t)\right|}{g(t)}\\ gMARD(\%)=\frac{1}{N}\sum_{t=1}^{N}\frac{Pen(g(t),\hat{g}(t))|g(t)-\hat{g}(t)|}{g(t)} \end{array}$$ (10)

The CEG [36] was included in the performance metrics to evaluate the clinical significance of the deviation of the estimated BG value from the target value. The CEG uses a Cartesian diagram on which the target and predicted BG values are paired. Each pair is located in one of five regions of the diagram. Region A contains those values within 20% of the reference sensor or pairs in which the predicted values and the reference values are <70 mg/dl. The pairs located in region A represent clinically correct predictions and, therefore, it is highly desirable to have all the results in this zone. Region B contains pairs by which therapy decisions made with an inaccurate estimate of the target value presents little danger. Region C contains pairs that lead to potentially dangerous overtreatment. Region D contains pairs that lead to missed severe episodes of hypoglycemia or hyperglycemia. Finally, region E contains the pairs of values that are the most different and yield the most erroneous predictions. Summarizing, pairs of points within regions A and B are clinically acceptable, while pairs in regions C, D and E are potentially dangerous and are considered significant clinical errors. Most of the CEG results are presented as a percentage of data that falls in each region relative to the total data for each case. The next subsections present and discuss the results of the evolving personalized models obtained using different approaches.

### Midterm BG prediction models

A personalized piecewise model was generated for each patient by dividing the days into four 6-h segments, with three segments involving a meal and a segment corresponding to the nocturnal period when no food is ingested. For this purpose, the algorithm incorporates the input and target values corresponding to a 6-h period and the outcome of the algorithm is set to a constant value for the timestamps that do not correspond to the segment of interest. Eq (11) presents an example of the 6-h breakfast model for Patient 2:

$$Pred_{G}(n)=[G(n)\;*Ra(n)-I_{OB}(n)]-Circadian(n)$$ (11)

where *n* represents the time step when the prediction is made and glucose, CHO, insulin, and circadian values are obtained via *G*, *I*_OB_, *R*_a_, and Circadian, which are defined in Eq (11) for the same example:

$$\begin{array}{c} G(n)=\frac{3.7\;log(G[n-24])}{57.9}+\frac{57.7}{{G[n-24]}^{2.0}}+\frac{909.1}{{G[n-24]}^{2.7}}\\ Ra(n)=-sin({G[n-24]}^{2.7})-90.0\\ I_{OB}(n)=2.0\;sin({G[n]}^{4.1})-\frac{4.9\;log\;({G[n-6]}^{4.9})}{0.2}\\ Circadian(n)=19.9\;sin((909.7\pi +5.5n/288)\pi ) \end{array}$$ (12)

**Fig 3:**
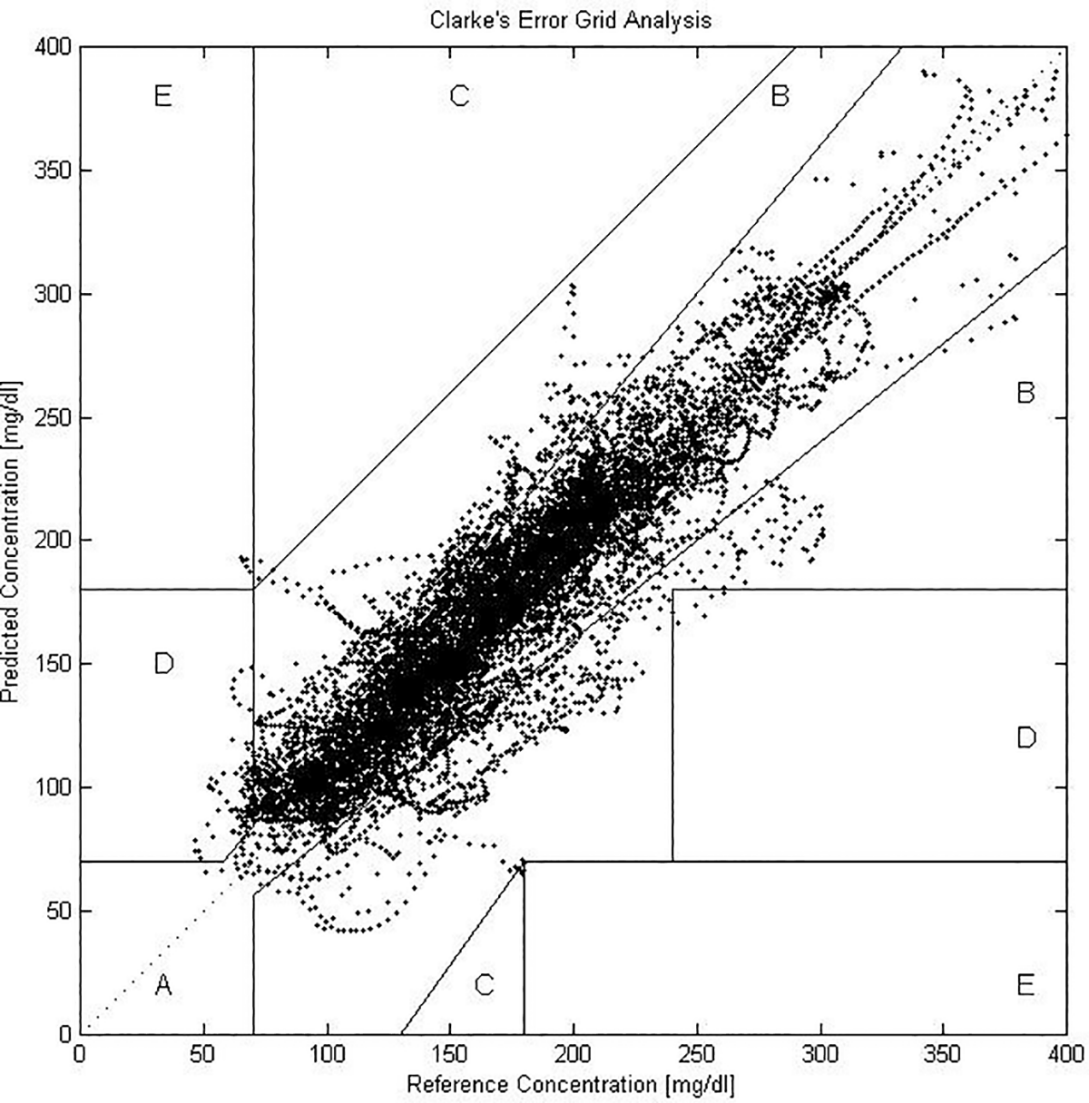
Clarke error grid for test data of 20 patients in the breakfast period.

Because the four periods of the day are each 6 h, the overall accuracy is the mean of the performance metrics for each portion of the day. Table 2 presents the averaged individual metrics for 100 patients for the four periods and the percentage distribution among the five CEG regions for the training and the test data. Because the four segments have the same amount of information, the overall performance metrics and the percentage distribution for the CEG for a 24-h model can be reported as average values in the last row of Table 2. In addition, to show the accuracy of the results, Fig 3 presents the CEG, i.e., the distribution of the prediction error based on its clinical harmfulness, for the test data of 20 patients in the breakfast scenario.

**Table 2:** Mean values of the performance metrics for 100 patients to fit 6-h prediction models.

Segment		RMSE (mg/dl)	gRMSE (mg/dl)	MAD (mg/dl)	gMAD (mg/dl)	MARD (%)	gMARD (%)	ZONE A+B (%)	ZONE A (%)	ZONE B (%)	ZONE C (%)	ZONE D (%)	ZONE E (%)
**Nocturnal**	Training	10.89	11.36	8.24	8.83	7.01	7.62	99.47	91.93	7.54	0.00	0.53	0.00
	Testing	11.80	12.19	9.10	9.68	7.62	8.25	99.37	90.53	8.84	0.00	0.63	0.00
**Breakfast**	Training	19.90	21.99	15.62	18.71	10.25	12.13	98.8	87.30	11.50	0.00	1.16	0.00
	Testing	22.09	24.60	17.39	21.11	11.50	13.78	98.68	83.38	15.30	0.00	1.26	0.00
**Lunch**	Training	18.33	19.90	14.45	16.58	11.12	12.97	98.32	84.50	13.82	0.01	1.66	0.00
	Testing	20.93	23.20	16.51	19.64	12.38	14.81	98.02	81.35	16.67	0.01	1.97	0.00
**Dinner**	Training	25.14	28.16	19.38	23.40	14.24	17.18	97.41	75.53	21.88	0.04	2.52	0.00
	Testing	29.00	33.00	22.80	27.90	16.00	19.80	97.16	70.66	26.50	0.22	2.65	0.01
**24 hour**	Training	18.57	20.35	14.42	16.88	10.66	12.48	98.51	84.82	13.69	0.01	1.47	0.00
	Testing	20.96	23.25	16.45	19.58	11.88	14.16	98.31	81.48	16.83	0.06	1.63	0.00

Our study also considered another perspective with respect to the use of the evolved personalized models. Table 3 presents the metrics results for models trained to perform 4-h postprandial predictions and thereby the deviation from the target values 2 h after the intake of the meal. Therefore, 100 individualized models were evolved to predict BG values using the same virtual cohort, albeit by optimizing the predictions for 4-h periods as specified in Table 3.

**Table 3:** Mean values of the performance metrics for 100 patients fitting 4-h prediction models.

Segment		RMSE (mg/dl)	gRMSE (mg/dl)	MAD (mg/dl)	gMAD (mg/dl)	MARD (%)	gMARD (%)	ZONE A+B (%)	ZONE A (%)	ZONE B (%)	ZONE C (%)	ZONE D (%)	ZONE E (%)
**09:00–13:00**	Training	16.14	17.56	12.67	14.8	8.99	10.59	98.5	89.33	9.17	0.00	1.50	0.00
	Testing	18.46	20.37	14.67	17.55	10.5	12.57	98.34	85.42	12.92	0.01	1.63	0.03
**15:00–19:00**	Training	14.99	16.02	11.86	13.34	9.93	11.63	97.89	86.54	11.35	0.00	2.11	0.02
	Testing	18.41	20.13	14.75	17.26	11.96	14.45	97.41	81.58	15.83	0.00	2.58	0.00
**19:00–01:00**	Training	19.53	21.45	15.26	17.96	12.33	14.98	96.87	81.09	15.78	0.04	3.09	0.00
	Testing	26.62	29.76	20.85	24.81	16.22	19.71	96.39	70.44	25.95	0.09	3.50	0.01

## Discussion

As observed in Table 2, the models produced adequate predictions for the nocturnal period in terms of RMSE, MAD, MARD, and their corresponding glucose-specific metrics gRMSE, gMAD, and gMARD, which was expected because of the lack of food intake. However, the results obtained for postprandial periods were not adequate. The dinner period models had the highest mean scores for the standard and glucose-specific metrics. Therefore, these models, on average, deviated the most from the reference values. However, even for this period, more than 97% of the prediction results fell inside regions A and B for the test data, which implies that the prediction was safe from a therapeutic point of view. Regarding the general distribution of the clinical harmfulness of the deviations, it is to be noted that most of the errors out of the zones A+B are concentrated in zone D. As previously stated in the performance metrics section, having pairs located in region D is highly undesirable because it means that the prediction missed a severe hypoglycemia or hyperglycemia state. Despite always being under 4% for all the scenarios, the use of the CEG led to the identification of a specific flaw in the personalized models, especially in the model including the last meal of the day, which is the possible underestimation of the BG concentrations during hyperglycemic events and overestimation of the BG levels during hypoglycemic events. This finding highlights the importance of the use of clinical harmfulness evaluation systems like CEG to identify poor performance in terms of relevant predictions like hypo/hyperglycemic events.

Since the dinner segment is, on average, the most challenging with respect to predictions, it likely would benefit from strategies designed specifically to improve the accuracy of the fitting process. For instance, Table 3 shows the results obtained after removing the first 2 h after meals from the fitting process because that period is unpredictable (at least a 120-min prediction horizon). The averaged performance metrics and the CEG percentages in Table 3 improved for the three meals compared with the results in Table 2. The relevance of this perspective concerns the applications in risk-based advisory systems or alarm systems that allow model training in dynamic response once blood glucose is expected to decay. Conversely, the full-segments perspective is justified by the empirical evidence, which shows that predicting the dynamics of the 2-h period immediately after meal intake is considerably more complex than predicting the BG level when the meal effect vanishes. To make decisions with respect to insulin therapy or the rescue of carbohydrate ingestion, the accuracy of the algorithm is favored by the evolution of specific prediction models for the postprandial period.

Antecedents of the approach presented in this paper are a study that assessed the feasibility of GE prediction systems based on a time series of historical prices [37], and the first study that adopted an approach toward personalized BG predictions using GE [38]. In contrast with previous GE approaches [22][23] that were limited to short-term predictions (>60 min) and were tested with five and eight virtual patients, respectively, obtained from the AIDA simulator, the present study was built and tested for midterm predictions (120 min) and in a more robust manner by using the UVA/Padova T1D Simulator [9], which described both intra- and interpatient glucose variability [26] implemented in the T1D patient decision-making model [39].

In general, recent studies usually predicted T1D glucose for the next 30 min [21]. However, some studies have reported on the evaluation of a 120-min prediction horizon. For instance, Georga et al. [40][41] assessed support vector regression and random forests methods using data from 15 patients. In both cases, the best performance metrics were obtained when using CGM (mg/dl), plasma insulin concentration (μU/ml), instantaneous energy expenditure, meal-derived glucose rate of appearance, and *R*_a_ (mg/min) as inputs. This resulted in an RMSE of 7.62 mg/dl using the SMV approach and 10.83 mg/dl using random forests. Likewise, Zarkogianni et al. [5] compared several data-based techniques using as inputs the most recent glucose measurement *G*(*t*), the change in glucose level Δ*G*(*t*), and the sum of energy expenditures during the last 30 min. This resulted in a technique based on the self-organizing map that yielded the most favorable results in terms of RMSE (31.00 ± 6.07) and MARD (14.56 ± 3.46) for a cohort of ten patients. Aside from using physical activity as an additional input signal, these approaches generated models that were trained and evaluated using the same data set and an independent data set was not used for validation.

Leal et al. [42] used support vector regression to predict nocturnal glucose, using CGM and insulin delivery information to produce individual models for the same 100 in silico T1D adults used in this study [9]. The results for four simulated night periods between 1 and 7 am were measured in terms of glucose-specific metrics. According to the means of those test metrics (RMSE = 15.0, gRMSE = 15.7, MAD = 10.9, gMAD = 11.7, MARD = 9.0, gMARD = 9.6), the predictions achieved by the proposed approach presented in this paper were more accurate. Moreover, in this paper, the clinical reliability of the predictions was evaluated in both the modeling process and the outcomes.

## Conclusion and future work

The results of this simulated study of both experimental approaches described in the previous section are promising. They confirm the assumption that the use of a glucose-specific cost function that takes into account the clinical harmfulness of deviations makes the prediction models more reliable in terms of clinical usefulness. In addition, these results suggest that dividing the day into different segments that can be studied separately and generating piecewise models improve the accuracy and clinical reliability of the overall model. These results also show that the performance and safety of the predictions can be improved further by generating a set of interchangeable models that predict useful BG values for control and therapy purposes based on the determination of individual specific dynamics, lifestyle, and other factors.

The ability to provide an early warning of ineffective or poor insulin treatment, which usually leads to hyperglycemic or hypoglycemic episodes, is of great interest. Useful real-time predictions of future CGM measurements are possible but challenging owing to various factors, including variability and the associated delays of food and insulin absorption. In addition, there is a 10–15-min lag between the actual blood plasma values and the sensor measurements, resulting in an approximate mean absolute relative difference (MARD) of 9% [3] for the best sensors.

Despite being limited by these delays, accurate forecasts can provide enough time to act in anticipation of the CGM measurements to prevent hyperglycemia or hypoglycemia. In this study, we developed a hybrid model that uses GE, insulin on board, and glucose rate of absorption models to predict BG values with a prediction horizon of 120 min. The algorithm relies on the construction of a set of rules that determine the search space for an optimization algorithm based on GE. A glucose-specific fitness function leads the evolution of the solution while penalizing deviations based on their clinical harmfulness and a tailored evolutionary grammar.

Our study proposed a hybrid GE and physiological model-based methodology for determining personalized midterm predictions of CGM readings. To the best of our knowledge, this is the first methodology to include physiological models in the overall GE model.

Future work will address the following points:

- The FDA-approved simulator used in this work is a valid substitute for the preclinical testing of novel technologies in diabetes care (see, e.g., [43][44][45]). However, the use of in silico data is not meant as substitute to human trial; rather, it can be considered a good starting point for evaluating our GE method: in fact simulated data are complete, i.e. there is no missing information related to meals, boluses, hypotreatments, or any other unexpected event. The natural extension of this work will be testing personalization of BG prediction models in a more challenging situation involving real subjects.
- Estimation of BG values can be automatically processed ahead of time to generate risk-based predictions. In addition, the risk of life-threatening events can be incorporated directly into the fitness function.
- There are few conclusive reports on exercise management with respect to the prediction model. We will examine the manner in which input signals related to physical exercise can improve or deteriorate the accuracy of personalized models.
- The grammar shapes the solution. For our approach, other grammar architectures can be explored to improve the accuracy and flexibility of the patient model. Future studies will include a comparative analysis that explores the decrease and increase in grammar complexity.
